# A Study of Mortality in Cardiac Patients in a Pediatric Intensive Care Unit

**DOI:** 10.7759/cureus.6052

**Published:** 2019-11-01

**Authors:** Zuhal Gundogdu, Kadir Babaoglu, Murat Deveci, Okan Tugral, Uyan ZS

**Affiliations:** 1 Pediatrics, Kocaeli University, Kocaeli, TUR; 2 Cardiology, Kocaeli University, Kocaeli, TUR

**Keywords:** mortality, congenital heart diseases, pim scores, pediatric intensive care

## Abstract

Objective: One of the major causes of mortality in the pediatric intensive care unit (PICU) is heart disease. This study aimed to determine the causes of mortality in children with pre-existing cardiac abnormalities who were admitted into the PICU.

Methods: Data were collected through patient profile assessment and outcome and heart diseases affecting prognosis were analyzed. Medical records of children were reviewed retrospectively. The updated Pediatric Index of Mortality 2 (PIM2) scores were used. Exploratory data analysis was performed using descriptive measures. Kolmogorov-Smirnov tests were used to test the normality of data distribution.

Results: Out of 566 admissions into PICU, 76 (13.4%) had cardiac abnormalities. Median and range of PICU stay were 5.50 and 417.88 days. The mean PIM2 score on admission was found to be 31.05. The most common admission was due to atrioventricular septal defect (AVSD) (15.7%), cardiomyopathy (13.1%), ventricular septal defect (VSD) (11.8%), tetralogy of Fallot (10.5%) and others (48.9%). There were multiple cardiac anomalies in 3.9% of patients. The most important cause of cardiac mortality in PICU was septic shock (26.0%) followed by cardiogenic shock (20.6%), and cardiac failure (13.7%). The nosocomial infection rate of cardiac patients in PICU was 10.5%.

Conclusions: Our study reconfirmed that the PIM2 score is a good indicator of cardiac diseases. Infections, nosocomial infections, pneumonia, and septic shock were the leading causes of mortality in cardiac patients. Better infection control in the PICU may have a significant impact on decreasing mortality rates.

## Introduction

Congenital heart defects (CHD) are serious and common conditions that have a significant impact on morbidity, mortality and healthcare costs in both children and adults [1]. Population-based epidemiological studies of CHD have indicated a prevalence ranging from 4.6 to 12.2 per 1,000 live births [2-6]. It is estimated that at least 32,000 infants in the United States will be affected each year by CHD [1]. Of these, approximately 25%, or 2.4 per 1,000 live births, require invasive treatment in the first year of life.

While advances in treatment in the last decades have decreased infant mortality, they have also led to an increase in the number of children and adults with CHD [7]. Despite these advances and developments in interventional and surgical techniques, heart disease in children continues to be an important cause of morbidity and mortality [8]. It has been reported that CHD was responsible for the largest proportion of mortality, (30%-50%) caused by birth defects among infants and young children [9]. However, with improved surgical techniques and post-operative care, survival rates do have a significant financial impact throughout the patient’s lifetime. These patients may require multiple surgeries and may be especially susceptible to infection from normal exposure to the general population, which may also lead to substantial subsequent mortality [10]. Advances in the medical and surgical management of CHD have improved survival rates and this has consequently resulted in a steady increase in the number of children with CHD surviving beyond infancy [11-14]. Despite recent developments in interventional and surgical techniques, heart diseases in children continue to be an important cause of morbidity and mortality [15-16]. Ventricular septal defect (VSD), pulmonary stenosis (PS), and atrial septal defect (ASD) have been reported as the most frequent CHDs [8].

This paper reports the causes of mortality in children with pre-existing cardiac abnormalities who were admitted into the pediatric intensive care unit (PICU).

## Materials and methods

Data were gathered from medical records of 566 children admitted into the PICU of Kocaeli University Hospital between December 2010 and January 2015. Medical records were reviewed retrospectively and 76 children with cardiac diseases were selected for inclusion in this study. Newborns were excluded from this study. This study was approved by the local hospital committee as, at the time of this study, no ethical approval was needed for retrospective studies.

Relevant data items were collected from the patient medical records. These included date of birth, gender, admission due to post-operative cardiac deterioration, PICU admission date, diagnosis, mechanic and/or nasal mechanical ventilation, hood or nasal oxygen, duration of ventilation, blood pressure and PICU discharge status (alive or deceased). Laboratory results such as initial C-reactive protein (CRP), the highest CRP, and CRP for hospital admissions longer than seven days, urine, blood, and endotracheal culture results were also noted. Reasons for admission, dates and number of cardiopulmonary resuscitations (CPRs), CPR success, and date of death were also recorded.

The updated Pediatric Index of Mortality 2 (PIM2) scores, which are calculated an hour after admission, were used as a measure of severity of the condition, risk assessment and death probability [17-19]. PIM2 score was calculated from the following data: elective admission, post-procedure recovery, cardiac bypass, high-risk diagnosis, low-risk diagnosis, pupil response to bright light, initial mechanical ventilation (any time during the first hour in PICU), initial systolic blood pressure, base excess (arterial or capillary blood), and FiO_2_×100/PaO_2_ [18-19].

Each child admitted into the PICU had a general examination by a pediatric consultant and cardiovascular examination by a pediatric cardiologist. All cases of heart abnormalities were detected by echocardiography at Kocaeli University Hospital. Echocardiography was conducted using M-mode, two-dimensional, and color, pulse and continuous wave Doppler echocardiogram. The presence and severity of any cardiac defect were analyzed as per recommendations of the American Society of Echocardiography [20]. Reasons for admission and causes of mortality in the PICU and any cases of infection or nosocomial infections were also recorded. Infections which, started seven days after admission into the PICU were defined as nosocomial infections.

Statistical Package for Social Sciences (SPSS), version 20 (IBM Inc., Chicago, IL) was used for statistical analysis. Exploratory data analysis was performed using descriptive measures. Kolmogorov-Smirnov tests were used to assess the normality of data distribution. Potentially confounding variables were evaluated with independent t-test or Mann Whitney test in order to explore the presence of suspected association of deceased and surviving patients.

## Results

Out of 566 admissions into the PICU during the study period, 76 out of 566 patients (13.4%) had cardiac abnormalities, thus the final study group included only 76 cardiac patients. The gender distribution of the study group was 32 (42.1%) male and 44 (57.9%) female. The age median and range of cardiac patients in the PICU was 0.50±16.98 years, respectively. Postoperative cardiac patients constitute 36.8% of the cardiac patients in PICU. Moreover, 27.6% of them were admitted into the PICU right after cardiac operation. Seventeen percent (17.1%) did not require mechanical ventilation. The median of PICU stay duration was 5.50±417.88 days (Table 1). The median of PIM2 score on admission was 31.05± 100. The most common admission was due to atrioventricular septal defect (AVSD) (15.7%), the rest are given in Table 1. Multiple cardiac anomalies were present in 3.9% of the patients. The survival rate was 61.8%.

**Table 1:** Cardiac Diseases in the Pediatric Intensive Care Unit (PICU)

Diagnosis	Number (%)
Tetralogy of Fallot (TOF)	8 (10.5)
Ventricular Septal Defect (VSD)	9 (11.8)
Truncus Arteriosus (TA)	1 (1.3)
Patent Ductus Arteriosus (PDA)	4 (5.2)
Mitral Regurgitation (MR)	2 (2.6)
Tricuspid Regurgitation (TR)	2 (2.6)
Atrioventricular Septal Defect (AVSD)	12 (15.7)
Atrial Septal Defect (ASD)	7 (9.2)
Pulmonary Stenosis (PS)	2 (2.6)
Total Anomalous Pulmonary Venous Return (TAPVR)	2 (2.6)
Coarctation of the Aorta (Coarc)	2 (2.6)
Hypoplastic Left Heart (HLH)	2 (2.6)
Arcus Aorta Anomaly	4 (5.2)
Transposition of the Great Arteries (TGA)	6 (7.8)
Aritmia	2 (2.6)
Cardiomyopathy	10 (13.1)
Pericardial anomaly	3 (3.9)
Ebstein anomaly	1 (1.3)
N=76	

There was a statistically significant difference in the length of the PICU stay, duration of mechanical ventilation, duration of nasal ventilation, duration of oxygen administration, PIM2 score, and number of CPR attempts, number of nosocomial infections and infection rates between deceased and surviving patients (Table 2).

**Table 2:** Analysis and Comparison of Deceased and Surviving Patients

		Deceased (N=29)	Surviving (N=47)	p
Age (in years) (Mean ± SD)		2.13± 3.60	2.62±4.84	1.000
Male (%)		12 (41.4%)	20 (42.6%)	0.180
Length of stay (in days) (Mean ± SD)		10.17±16.15	27.06±69.91	0.002
Mechanical ventilation (in days) (Mean ± SD)		8.03±12.22	21.31±66.20	0.000
Nasal mechanical ventilation (in days) (Mean ± SD)		0.10±0.55	1.80±4.52	0.000
O_2_ administration (in days) (Mean ± SD)		1.68±4.48	4.10±5.89	0.000
				0.000
Number of CPR attempts per child	0	0	45 (95.7)	
	0-3	28 (96.5)	2 (4.2)	
	>3	1 (3.4)		
PIM2 score (Mean ± SD)		88.67±13.73	46.20±34.31	<0.001
Infection rate (%)		41.4%	29.8%	0.011
Nosocomial infection rate (%)		17.2%	6.4%	0.002

These two groups were similar with respect to age and gender. The infection rate was 41.4% in the follow-up period among the patients who subsequently died. Table 3 shows the major causes of mortality in cardiac patients.

**Table 3:** Mortality Reasons for Cardiac Patients in the Pediatric Intensive Care Unit (PICU)

Deceased Patients	Total (N=29)
Pneumonia (%)	12.3%
Embolism (%)	3.4%
Cardiac failure (%)	13.7%
Acute respiratory distress syndrome (ARDS) (%)	6.9%
Septic shock (%)	26.0%
Cardiogenic shock (%)	20.6%
Arrhythmia (%)	3.4%
Myocarditis (%)	3.4%
Infective endocarditis (%)	6.9%
Bronchopulmonary dysplasia (BPD)	3.4%

## Discussion

Results of a study on CHDs show similar distributions [15]. In our study, VSD (22.2%) was the most frequent congenital heart disease, followed by ASD (11.1%), patent ductus arteriosus (PDA) (9.2%), and PS (4.4%). The most common admission into the PICU was for AVSD and cardiomyopathy in our study. However, one should note that the number of patients in our study is not sufficient to show these results as the main incidence of heart diseases.

Gilboa et al. (2010) reported that CHD mortality is highest among infants and lowest among children aged between 5 and17 years [9]. This study showed that nearly half of all mortality caused by CHD occurred during infancy. In contrast, our results have shown that the mean of mortality age of cardiac patients was nearly two years, but the ages of deceased and surviving cardiac patients were not different in the PICU. It was reported that the CHD mortality rate was significantly lower among male patients [9]. However, our study shows that survival rates were similar in both female and male PICU patients. PIM2 scores, which are used as a measure of illness severity and predict the probability of death, are important criteria and there was a significant difference in PIM2 scores between deceased and surviving patients in our study.

We have shown that infection, especially septic shock, was an important cause of the mortality due to heart disease in PICU: The infection rate was 41.4% among deceased cardiac patients, showing this to be a major mortality cause, followed by cardiogenic shock and cardiac failure. The nosocomial infection rate among deceased cardiac patients was 17.2% in PICU. Over 50% of hospital admissions related to acute respiratory tract infections in children with CHD are due to bronchiolitis [16]. High levels of near-capacity demand in the winter period, when respiratory admissions are at their highest, show increased mortality in children admitted into PICUs at this time [16]. The pneumonia rate among deceased cardiac patients was 12.3% in our study. Although this study is not large enough to state this as a prevalence study, it might still be useful to decrease the mortality rate due to heart diseases in PICU.

Infection, especially septic shock was an important reason for the mortality of CHD patients in PICU. The infection rate was 41.4% among deceased cardiac patients showing this to be a major mortality cause, followed by cardiogenic shock and cardiac failure. The nosocomial infection rate among the deceased cardiac patients was 17.2%.

## Conclusions

CHD is a common reason for PICU admissions. Taking more preventive measures against infections in patients with known CHD may reduce the morbidity and mortality rates. Our study reconfirmed that PIM2 score is a good indicator of the mortality of cardiac patients. Infections and nosocomial infections were quite high and pneumonia was the leading cause of mortality in cardiac diseases. Better control of infection leads to better survival rates in CHD patients.
